# Tolerance to Ethanol or Nicotine Results in Increased Ethanol Self-Administration and Long-Term Depression in the Dorsolateral Striatum

**DOI:** 10.1523/ENEURO.0112-15.2016

**Published:** 2016-08-04

**Authors:** Chandrika Abburi, Shannon L. Wolfman, Ryan A. E. Metz, Rinya Kamber, Daniel S. McGehee, John McDaid

**Affiliations:** 1 Department of Anesthesia and Critical Care, University of Chicago, Chicago, Illinois 60637; 2 Committee on Neurobiology, University of Chicago, Chicago, Illinois 60637

**Keywords:** addiction, reward, endocanabinoid, motor impairment, self-administration

## Abstract

Ethanol (EtOH) and nicotine are the most widely coabused drugs. Tolerance to EtOH intoxication, including motor impairment, results in greater EtOH consumption and may result in a greater likelihood of addiction. Previous studies suggest that cross-tolerance between EtOH and nicotine may contribute to the abuse potential of these drugs. Here we demonstrate that repeated intermittent administration of either EtOH or nicotine in adult male Sprague Dawley rats results in tolerance to EtOH-induced motor impairment and increased EtOH self-administration. These findings suggest that nicotine and EtOH cross-tolerance results in decreased aversive and enhanced rewarding effects of EtOH. Endocannabinoid signaling in the dorsolateral striatum (DLS) has been implicated in both EtOH tolerance and reward, so we investigated whether nicotine or EtOH pretreatment might modulate endocannabinoid signaling in this region. Using similar EtOH and nicotine pretreatment methods resulted in increased paired-pulse ratios of evoked EPSCs in enkephalin-positive medium spiny neurons in DLS slices. Thus, EtOH and nicotine pretreatment may modulate glutamatergic synapses in the DLS presynaptically. Bath application of the CB1 receptor agonist Win 55,2-212 increased the paired-pulse ratio of evoked EPSCs in control slices, while Win 55,2-212 had no effect on paired-pulse ratio in slices from either EtOH- or nicotine-pretreated rats. Consistent with these effects, nicotine pretreatment occluded LTD induction by high-frequency stimulation of the corticostriatal inputs to the dorsolateral striatum. These results suggest that nicotine and EtOH pretreatment modulates striatal synapses to induce tolerance to the motor-impairing effects of EtOH, which may contribute to nicotine and EtOH coabuse.

## Significance Statement

This study demonstrates that repeated intermittent nicotine or ethanol pre-exposure results in lower levels of ethanol-induced motor impairment and higher levels of ethanol self-administration. These effects of pretreatment suggest cross-tolerance between these drugs, which may contribute to the development of dependence. These studies identify cellular mechanisms underlying the development of ethanol tolerance that may lead to novel treatments for alcohol and nicotine dependence.

## Introduction

Ethanol (EtOH) and nicotine addiction are two of the leading causes of preventable death worldwide. These are the most commonly coabused drugs, with a large majority of alcoholics diagnosed with a comorbid addiction to nicotine (Miller and Gold, 1998). In fact, alcoholics who are also smokers drink more, have stronger cravings, and are more severely alcohol dependent (Batel et al., 1995; York and Hirsch, 1995; Glautier et al., 1996; Daeppen et al., 2002; Rose et al., 2002; Sayette, 2002; John et al., 2003; Hertling et al., 2005; Acheson et al., 2006; Barrett et al., 2006; King et al., 2010; Buu et al., 2014; McClure et al., 2015). Many factors likely contribute to the prevalence of EtOH and nicotine coabuse, and understanding the neurobiological underpinnings may help to identify novel treatments for addiction to these two drugs.

Cross-tolerance to the aversive effects of EtOH and nicotine likely facilitates the coabuse of these drugs, as nicotine enhances the rewarding effects of EtOH, while attenuating some of the more negative sedative and cognitive effects (Perkins et al., 1995; Collins et al., 1996b; Söderpalm et al., 2000; Schuckit et al., 2001; Johnson, 2004; Ceballos, 2006; Funk et al., 2006; Morrow et al., 2006). Numerous studies have examined cross-tolerance between EtOH and nicotine; however, the behavioral tasks, drug doses, and administration methods vary widely (Potthoff et al., 1983; Collins et al., 1988; Burch et al., 1988; Blomqvist et al., 1996; López-Moreno et al., 2008; Biała and Budzyńska, 2010; Lê et al., 2014). We know that exposure to EtOH or nicotine alters neural circuitry underlying reward and sedation/cognition, but a link between these two behavioral end points in the context of cross-tolerance has not been established. Therefore, we investigated the effects of cross-tolerance between ethanol and nicotine on both reward- and sedation-related behaviors.

In humans, smoking history can predict future EtOH dependence (John et al., 2003; Buu et al., 2014), which suggests that previous exposure to nicotine impacts the behavioral effects of EtOH, even in the absence of concurrent nicotine exposure. For our studies, moderate, physiologically relevant doses of both drugs were used to test the hypothesis that nicotine pre-exposure would enhance EtOH self-administration and decrease EtOH-induced motor impairment. We also predicted that a common neurobiological change would accompany these behavioral changes.

As the dorsolateral striatum (DLS) is involved both in the rewarding and motor effects of EtOH and nicotine (Meng et al., 1998; Garção et al., 2013; Chen et al., 2014), we focused our electrophysiology investigations on this brain region. EtOH and nicotine modulate endocannabinoid signaling in the DLS (González et al., 2002; Marco et al., 2007; Adermark et al., 2011; Vinod et al., 2012; DePoy et al., 2013, 2015), and CB1 receptor agonists produce cross-tolerance with both EtOH and nicotine (Sprague and Craigmill, 1976; Siemens and Doyle, 1979; da Silva et al., 2001; Valjent and Mitchell, 2002; Lemos et al., 2007; Biała and Budzyńska, 2010). Additionally, the administration of either EtOH or nicotine results in increased endocannabinoid release and decreased CB1 receptor expression (Basavarajappa et al., 1998; Hungund and Basavarajappa, 2000; González et al., 2002; Marco et al., 2007; Adermark et al., 2011; Vinod et al., 2012; DePoy et al., 2013). Therefore, we investigated the effects of both EtOH and nicotine pretreatment on cannabinoid signaling in the DLS, and how these effects correlate with EtOH reward and motor impairment.

## Materials and Methods

### Animals

Adult male Sprague Dawley rats [postnatal day 60 (P60) to P90; Harlan] were housed two per cage with a 12 h reverse light/dark cycle and *ad libitum* access to food and water. During EtOH self-administration, rats were singly housed. All animal procedures were performed in accordance with the regulations of the University of Chicago animal care committee.

### Drugs and reagents

All drugs and reagents were obtained from Sigma-Aldrich, unless otherwise noted. Nicotine hydrogen tartrate salt was used for nicotine treatments, and 99% ethanol was used for EtOH treatments, as described in greater detail below.

### Rotarod testing

On training day, rats were placed on the rotarod (Rotamex 5, Columbus Instruments) at a fixed speed of 4 rpm. After each animal demonstrated an ability to stay on the rotarod at this speed for ∼10 s, the speed was increased at a rate of 1 rpm every 5 s until the last animal fell off. This protocol was repeated for a total of 10 consecutive trials, after which the animals were placed back in their home cages. Three days later, animals were tested for rotarod performance. Over a set of four consecutive trials, baseline performance was assessed. Immediately following completion of the baseline trials, rats were injected with either nicotine (0.1 mg/kg, s.c., as base), vehicle (PBS, 1 ml/kg, s.c.), or EtOH (1 g/kg, i.p., 50% in PBS), depending on the experiment. Fifteen minutes after injection, rotarod performance was again tested over a set of four consecutive trials. A total of six sets of four trials were conducted at 15 min intervals, including the baseline trials. For experiments with repeated rotarod testing, the same protocol was used for the next 2 d. On the last day, the same protocol was used, but all rats received EtOH injections.

### Home-cage pretreatment

Rats received injections of either PBS (1 ml/kg, s.c.), nicotine (0.1 mg/kg, s.c., as base, once per day), or EtOH (1 g/kg, s.c., 50% in PBS, twice per day 4 h apart) for 3 consecutive days. EtOH injection schedule was chosen to ensure several hours of a moderate blood EtOH concentration. Animals in the rotarod experiments were given home-cage injections 3 d after rotarod training, and rotarod testing commenced the day following the final home-cage injection. Animals in the self-administration experiments were given access to the two-bottle choice test the day following the final home-cage injection. Animals used for slice experiments were killed the day following the final home-cage injection.

### EtOH self-administration

Rats were singly housed for 3 d before receiving home-cage injections. The day after the final injection, rats were given continuous access to two drinking bottles in the home cage. One contained water, and the other contained 20% EtOH (v/v) in water. Water and EtOH consumption were measured every 48 h by weighing the bottles. The bottles were switched between sides after every measurement in order to control for side preferences. Self-administration continued for 20 d.

### Electrophysiology

The day following the final home-cage injection, rats were decapitated under isoflurane anesthesia, and brains were removed and transferred into ice-cold NMDG solution (in mm: *N*-acetyl-cysteine 12, NMDG 93, KCl 2.5, NaH_2_PO_4_ 1.2, NaHCO_3_ 30, HEPES 20, glucose 25, sodium ascorbate 5, thiourea 1.97, sodium pyruvate 3, MgSO_4_(7H_2_0) 10, CaCl_2_ (2H_2_0) 0.5, pH 7.4 with HCl; bubbled continuously with 95% O_2_/5% CO_2_). The 250 μm coronal slices containing the striatum were obtained with vibrating blade microtome (VT1000 S, Leica) in NMDG protective slicing solution (Zhao et al., 2011). Slices were transferred to an NMDG-containing holding chamber and allowed to recover for 10 min at 32°C. The slices were then moved to a holding chamber perfused with modified HEPES holding aCSF (in mm: N-acetyl-cysteine 12, NaCl 92, KCl 2.5, NaH_2_PO_4_ 1.2, NaHCO_3_ 30, HEPES 20, glucose 25, sodium ascorbate 5, thiourea 1.97, sodium pyruvate 3, MgSO_4_(7H_2_0) 2, CaCl_2_(2H_2_0) 2, pH 7.4 with NaOH; bubbled continuously with 95% O_2_/5% CO_2_) at a rate of 20 ml/min for at least 30 min at 32°C. For recording, slices were transferred to a recording chamber superfused with aCSF (in mm: NaCl 125, KCl 2.5, MgCl_2_ 1, CaCl_2_ 2.5, glucose 20, NaH_2_PO_4_ 1, NaHCO_3_ 25, ascorbic acid 1; bubbled with 95% O_2_/5% CO_2_) at a rate of 2 ml/min. Recordings were performed at room temperature (RT). The dorsolateral striatum was identified according to Paxinos and Watson (1998), and medium spiny neurons (MSNs) were visualized under infrared illumination using an upright microscope (BX51WI, Olympus). Standard whole-cell voltage-clamp recordings used a multiclamp 700B amplifier, a Digidata 1440 interface, and Clampex version 10.4 software (Molecular Devices). All recordings were filtered at 1 kHz and digitized at 5 kHz, *V*_m_ = −70 mV. For all recordings, we used borosilicate electrodes pulled to a resistance of 3-7 MΩ and containing a recording solution consisting of (in mm), cesium gluconate 117, HEPES 20, EGTA 0.4, NaCl 2.8, ATP 2.5, GTP 0.25, glucose 20, TEA 5, QX314 5, biocytin, 0.01% (AnaSpec, Inc.; pH 7.4 with CsOH). Series resistance was <20 MΩ, cells that reached higher resistances during the course of recording were discarded. All recordings were conducted in the presence of 20 µm bicuculline (Tocris Bioscience) to limit indirect effects from GABAergic synaptic inputs. EPSCs were evoked during recording from MSNs using a bipolar platinum-iridium-stimulating electrode placed inside the cortical border of the DLS. This placement favors the activation of corticostriatal synaptic inputs.

#### Paired-pulse ratios

To obtain paired-pulse ratios, we used a 50 ms interstimulus interval between evoked EPSCs, and the ratio was calculated as the second EPSC amplitude/the first EPSC amplitude (P2/P1). To compare the effect of *in vivo* pretreatment with nicotine or EtOH, three consecutive paired-pulses were obtained at 1 min intervals and averaged for each neuron tested. For testing the CB1 agonist, we obtained paired-pulse EPSCs at 1 min intervals until 5 min of consistent P2/P1 ratios were observed. The CB1 agonist Win 55,2-212 (Tocris Bioscience) was then bath applied at a concentration of 5 µm, and the P2/P1 ratio was monitored at 1 min intervals for at least 20 min.

#### HFS-induced long-term depression

Long-term depression (LTD) in MSNs was induced by stimulating corticostriatal glutamatergic fibers with high-frequency stimulation (HFS) consisting of a single train, with 1 s duration and 100 Hz frequency, after 5–10 min of stable baseline recordings. *V*_m_ = −70 mV throughout the recording and HFS stimulation. Average EPSC amplitudes from 20-30 min post-HFS were normalized to the average of EPSCs recorded during the 10 min baseline period for each neuron tested. These normalized values from nicotine- and vehicle-pretreated animals were pooled and compared using an unpaired *t* test.

### Immunohistochemistry

The internal solution used during whole-cell recordings contained biocytin (0.01%) for *post hoc* identification of the recorded cells. Enkephalin immunostaining allowed for the determination of the MSN subtype from which the recording was done (McGinty, 2007). Slices were fixed in 4% paraformaldehyde overnight after recording. Fixed slices were washed three times with 1× PBS (each wash for 5 min) and then blocked in 1× PBS containing 1% Triton X-100, 10% normal donkey serum, and 1% bovine serum albumin for 30 min at RT. The slices were incubated with 1° goat pAb to enkephalin (1:200; ab77273, Abcam) in blocking solution overnight at RT. Following three washes in 1× PBS, slices were incubated with Alexa Fluor 488 Donkey anti-goat IgG (1:1000; Invitrogen) and streptavidin Alexa Fluor 594 conjugate (1:1000; Invitrogen) for 3 h at room temperature. Finally, slices were washed three times with PBS, mounted, and coverslipped with Fluoromount-G (Southern Biotech). Stained slices were imaged under a fluorescent microscope to determine colocalization of enkephalin and biocytin.

### Data analysis and statistics

All electrophysiology data was collected using Clampex version 10.4 (Molecular Devices). Evoked EPSCs were analyzed using Clampfit (Molecular Devices). Two-way repeated-measures (RM) ANOVA followed by a Holm–Sidak *post hoc* test was used to determine the effects of PBS/nicotine/EtOH pretreatments on behavioral tasks. One-way ANOVA was used to analyze the effects of pretreatments on EPSC amplitudes and the paired-pulse ratios of evoked EPSCs. Paired *t* test was used for analysis of CB1 agonist effects; comparison was made between the baseline for each treatment group and the effects of the agonist. An unpaired *t* test was used to assess differences in LTD induction between nicotine- and vehicle-treated rats. All statistical tests were performed using SigmaPlot (Systat), and all results are presented as the mean ± SEM.

## Results

### Tolerance to EtOH increases EtOH self-administration

**Fig. 1.**
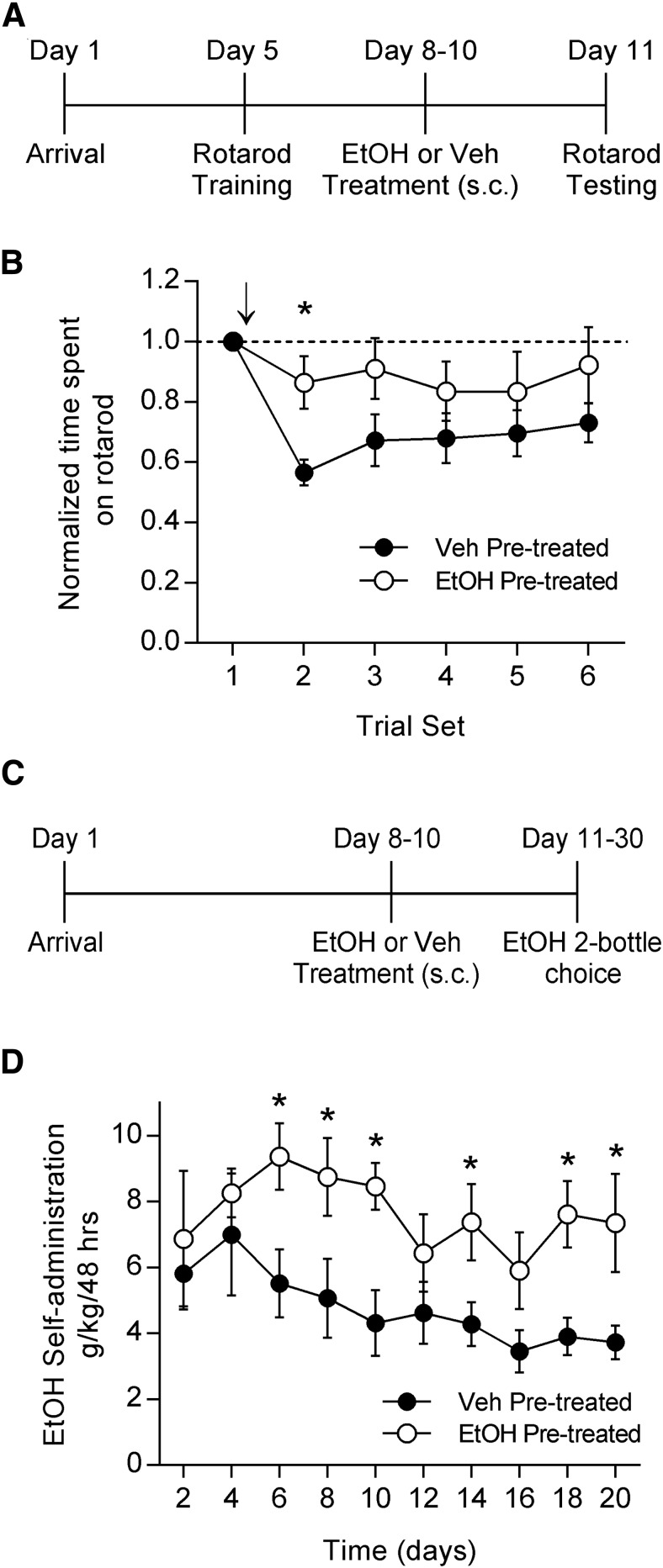
EtOH pretreatment results in tolerance to EtOH-induced motor impairment and increased EtOH self-administration. ***A***, Timeline for the rotarod experiment in which rats were pretreated in the home cage with either EtOH or vehicle. ***B***, Animals previously trained on the accelerating rotarod and pretreated for 3 d with either EtOH (1 g/kg, s.c., twice per day) or vehicle were tested for the effects of EtOH (1 g/kg, i.p.) on rotarod performance. Arrow indicates the time at which acute EtOH was administered. EtOH-pretreated animals displayed less impairment on the rotarod after acute EtOH administration than did the vehicle-treated animals (ANOVA, *p* = 0.0664; Holm–Sidak *post hoc* test, *p < 0.05; EtOH, *n* = 6; vehicle, *n* = 7). ***C***, Timeline for the EtOH two-bottle choice self-administration experiment in which rats were pretreated in the home cage with either EtOH or vehicle. ***D***, Animals pretreated with EtOH (1 g/kg, s.c., twice per day) or vehicle were given 24 h access to both a bottle of water and a bottle of 20% EtOH for 20 d. EtOH pretreated animals self-administered more EtOH than vehicle-pretreated animals (ANOVA, *p* < 0.05; Holm–Sidak *post hoc* test, *p < 0.05; *n* = 8 for both groups).

Motor impairment is a key adverse effect of EtOH, and tolerance to this aversive effect may promote escalated drinking. We first wanted to determine whether EtOH pretreatment in the home cage was sufficient in our hands to produce tolerance to the motor-impairing effects of EtOH. We used a physiologically relevant dose of EtOH (1 g/kg, i.p., 50% in PBS) and administered either EtOH or vehicle (PBS) twice per day for 3 d. We then used an accelerating rotarod to test motor performance in response to a challenge dose of EtOH (1 g/kg, i.p.; Fig. 1*A*). Because we were interested in examining the effects of EtOH pretreatment on rotarod performance rather than learning, we trained the rats on the rotarod prior to the first exposure to EtOH. Training consisted of 10 consecutive trials on the rotarod during 1 d. This protocol resulted in a consistent level of baseline performance on the testing day.

On testing day, the rats were subjected to one set of four consecutive trials to establish baseline performance (trial set 1). Animals then received a challenge injection of EtOH and were tested in five additional sets of trials. Trial 2 began 15 min after EtOH administration, and subsequent trials were performed in 15 min intervals. EtOH pretreatment in the home cage resulted in decreased motor impairment in response to an acute EtOH challenge compared with vehicle pretreatment (Fig. 1*B*; two-way RM ANOVA, *p* = 0.0664 time × treatment interaction; Holm–Sidak test for multiple comparisons, *p* = 0.0119; EtOH group, *n* = 6; PBS group, *n* = 7). This suggests that this limited, intermittent EtOH pretreatment was sufficient to produce tolerance to the motor-impairing effects of acute EtOH. This experiment confirmed past findings of tolerance to the motor-impairing effects of EtOH (Siemens and Doyle, 1979; Batista et al., 2005; Werner et al., 2009) and provided a baseline to which we could compare the possible effects of EtOH-nicotine cross-tolerance on the motor-impairing effects of EtOH.

We then tested whether the same EtOH pretreatment that produces tolerance to EtOH would also be sufficient in our hands to increase EtOH self-administration. We singly housed the rats and used the same dose and schedule of EtOH pretreatment. We then gave the rats 24 h access to two bottles, one that contained water, and one that contained 20% EtOH during the two-bottle choice self-administration procedure for 20 d (Fig. 1*C*). EtOH and water consumption were measured every 48 h by weighing the bottles. The sides on which the bottles were placed were alternated after every measurement to avoid the potential confound of a side preference. EtOH pretreatment resulted in increased EtOH self-administration compared with vehicle pretreatment (Fig. 1*D*; two-way RM ANOVA, *p* = 0.011 between treatments; Holm–Sidak test for multiple comparisons: day 6, *p* = 0.014; day 8, *p* = 0.018; day 10, *p* = 0.008; day 14, *p* = 0.045; day 18, *p* = 0.017; day 20, *p* = 0.02; *n* = 8 in each group). These results are consistent with previous findings that EtOH pretreatment enhances EtOH self-administration (Deutsch and Koopmans, 1973; Fidler et al., 2011). However, we used lower, more physiologically relevant doses of EtOH than have most prior studies, and we administered EtOH less frequently. Additionally, our data suggest that tolerance to EtOH can contribute to enhanced EtOH intake.

### Nicotine pretreatment results in tolerance to EtOH-induced motor impairment and increased EtOH self-administration

**Fig. 2.**
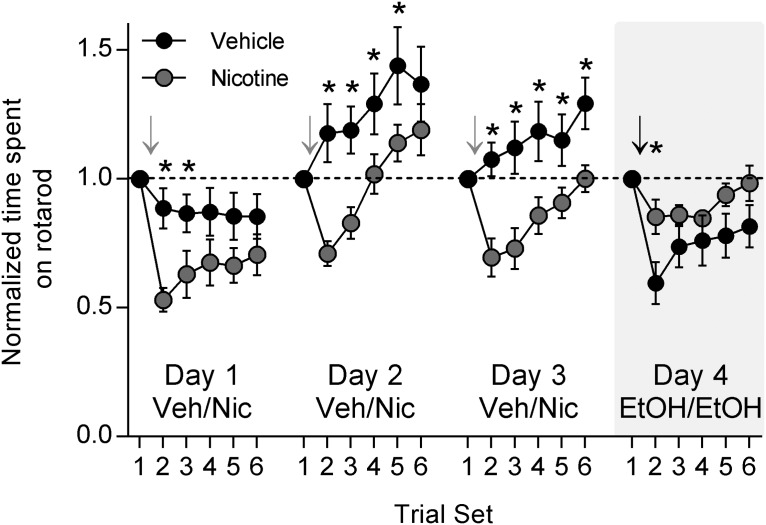
Acute nicotine administration causes motor impairment, but repeated administration results in tolerance to EtOH-induced motor impairment. Rats that were previously trained on the rotarod were tested for motor performance over 4 d. For the first 3 d, rats were administered either nicotine (0.1 mg/kg, s.c.) or vehicle. Gray arrows indicate the time point at which nicotine was administered. On the fourth day, both nicotine- and vehicle-treated groups were challenged with EtOH and tested for motor performance on the rotarod. The black arrow indicates the time at which EtOH was given. Acute nicotine administration resulted in significant motor impairment over all 3 d compared with vehicle administration (ANOVA, *p* < 0.0001 days 1, 2, 3; Holm–Sidak *post hoc* test, *p < 0.05; nicotine: *n* = 8, vehicle: *n* = 7). Repeated nicotine administration, however, resulted in tolerance to EtOH-induced motor impairment compared with vehicle administration (ANOVA, *p* < 0.001; Holm–Sidak *post hoc* test, *p < 0.05).

To explore the effects of nicotine on EtOH-induced motor impairment, we again used the accelerating rotarod. We were also interested in testing the acute effects of nicotine on rotarod performance. After training on the rotarod was completed, rotarod testing began in conjunction with daily nicotine treatments. After the baseline trial set, nicotine (0.1 mg/kg, s.c.) or vehicle was administered. After 15 min, trial set 2 began, and subsequent trial sets continued at 15 min intervals. This combination of nicotine or vehicle administration and rotarod testing was performed for 3 d. Acute nicotine administration caused impairment on the rotarod compared to vehicle administration on all 3 d (Fig. 2; two-way RM ANOVA, day 1, 2, 3: *p* < 0.0001 between treatments; Holm–Sidak test for multiple comparisons: day 1 trial set 2, *p* = 0.006; day 2 trial set 2, *p* = 0.003; day 2 trial set 3, *p* = 0.0314; day 3 trial set 2, *p* = 0.003; trial set 3, *p* = 0.0027; trial set 4, *p* = 0.012; trial set 5, *p* = 0.0499; trial set 6, *p* = 0.0223; nicotine group, *n* = 8; vehicle group, *n* = 7). On day 4, both nicotine- and vehicle-treated rats were given a challenge EtOH dose (1 g/kg, i.p.) after the baseline trial sets. Rotarod testing continued as usual at 15 min intervals. Animals that had received nicotine treatments on days 1–3 displayed significantly less EtOH-induced motor impairment than did animals that had received vehicle treatments (Fig. 2; two-way RM ANOVA, *p* = 0.0004 between treatments; Holm–Sidak test for multiple comparisons, *p* = 0.0268). These data show that nicotine is not simply enhancing performance on the rotarod and thus offsetting the motor-impairing effects of EtOH. They also show that nicotine treatment does not seem to impact the rate of improvement of rotarod performance. Together, our findings suggest that nicotine pretreatment, in the absence of concomitant drug exposure, results in cross-tolerance with EtOH and that cross-tolerance between EtOH and nicotine attenuates EtOH-induced motor impairment.

**Fig. 3.**
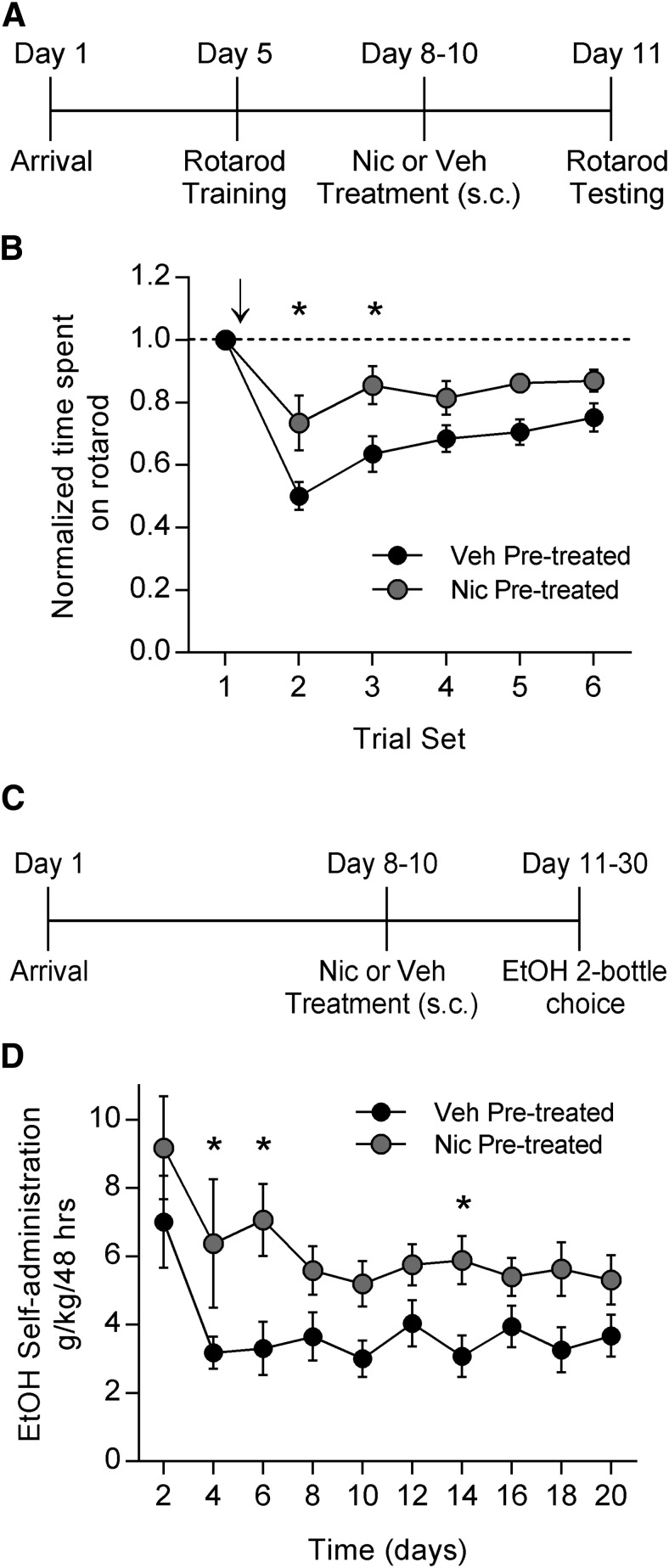
Nicotine pretreatment results in tolerance to EtOH-induced motor impairment and increased EtOH self-administration. ***A***, Timeline for the rotarod experiment in which rats were pretreated with either nicotine or vehicle. ***B***, Animals previously trained on the accelerating rotarod and pretreated for 3 d with either nicotine (0.1 mg/kg, s.c., once per day) or vehicle were tested for the effects of EtOH (1 g/kg, i.p.) on rotarod performance. Arrow indicates time at which acute EtOH was administered. Nicotine pretreated animals displayed less impairment on the rotarod after acute EtOH administration than did the vehicle-treated animals (ANOVA, *p* < 0.05; Holm–Sidak *post hoc* test, *p < 0.01; *n* = 14 for each group). ***C***, Timeline for the EtOH two-bottle choice self-administration experiment in which rats were pretreated with either nicotine or vehicle. ***D***, Animals pretreated with nicotine (0.1 mg/kg, s.c., once per day) or vehicle were given 24 h access to both a bottle of water and a bottle of 20% EtOH for 20 d. Nicotine-pretreated animals self-administered more EtOH than vehicle-pretreated animals (ANOVA, *p* < 0.01; Holm–Sidak *post hoc* test, *p < 0.05; *n* = 8 for both groups).

To further investigate this cross-tolerance without the confound of repeated rotarod testing, we trained animals on the rotarod and then pretreated with either nicotine (0.1 mg/kg, s.c., as base) or vehicle once per day for 3 d. The day after the last injection, rotarod testing began, and both groups were given a challenge dose of EtOH (1 g/kg, i.p.) after baseline testing (Fig. 3*A*). Nicotine-pretreated animals were significantly less impaired on the rotarod in response to EtOH than were vehicle-pretreated animals (Fig. 3*B*; two-way RM ANOVA, *p* = 0.0358 time × treatment interaction; Holm–Sidak test for multiple comparisons: trial set 2, *p* = 0.0038; trial set 3, *p* = 0.0063; *n* = 14 in each group). Nicotine pretreatment results in tolerance to EtOH-induced motor impairment.

Next, we investigated whether this regimen of nicotine pretreatment would also enhance EtOH self-administration. After singly housing the rats, nicotine or vehicle pretreatment took place once per day for 3 d. The rats were then given 24 h access to a water bottle and a bottle of 20% EtOH, as in the previous two-bottle choice paradigm, for 20 d (Fig. 3*C*). Nicotine pretreatment resulted in enhanced EtOH self-administration compared with vehicle pretreatment (Fig. 3*D*; two-way RM ANOVA, *p* = 0.005 between treatments; Holm–Sidak test for multiple comparisons: day 4, *p* = 0.011; day 6, *p* = 0.003; day 14, *p* = 0.025; nicotine group, *n* = 16; vehicle group, *n* = 15). Note that the vehicle-pretreated groups in the ethanol experiments displayed higher EtOH self-administration during the first 10 d relative to the control animals in the nicotine experiment. This is most likely due to differences in stress, as the EtOH experiment required twice-daily injections, while the nicotine experiment involved injections only once per day. Previous studies have reported stress-induced enhancement of EtOH self-administration (Meyer et al., 2013), but the important observation here is that both EtOH and nicotine pretreatment elevated EtOH self-administration relative to control treatments.

### EtOH and nicotine pretreatment similarly modulate DLS cannabinoid signaling

**Fig. 4.**
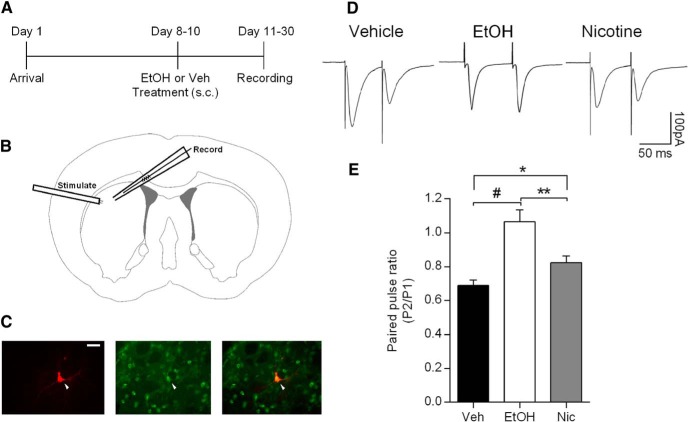
EtOH and nicotine pretreatment increase the paired-pulse ratio in enkephalin-positive (putative D_2_ receptor-containing) MSNs in the DLS. ***A***, Timeline for electrophysiology experiments in which rats were pretreated with EtOH (1 g/kg, s.c., twice per day), nicotine (0.1 mg/kg, s.c., once per day), or vehicle. ***B***, Schematic showing the positioning of recording and stimulating electrodes in the DLS. ***C***, Images of MSNs in the DLS. Arrowheads show the neuron that was recorded from. From left to right: representative biocytin-filled MSN (red); immunohistochemical labeling of enkephalin-positive MSNs (green); merged image (scale bar, 50 μm). All neurons included were putative D_2_ MSNs. ***D***, Representative traces showing the effects of vehicle, EtOH, or nicotine pretreatments on the paired-pulse ratio of EPSCs. ***E***, Summary of the effects of vehicle, EtOH, or nicotine pretreatment on paired-pulse ratios in putative D_2_-containing MSNs. EtOH and nicotine pretreatment both increased the paired-pulse ratio compared with vehicle pretreatment (ANOVA, *p* < 0.0001; Holm–Sidak *post hoc* test, **#***p* < 0.0001, *p < 0.05), and EtOH pretreatment increased the paired-pulse ratio significantly more than nicotine pretreatment (**p < 0.01; vehicle: *n* = 19; EtOH: *n* = 16; nicotine: *n* = 23; one cell/slice/rat).

Because EtOH and nicotine have both been reported to impact cannabinoid signaling in the DLS, we wanted to investigate and compare the effects of our EtOH and nicotine pretreatment regimens on DLS synapses. We used slice electrophysiology to examine release probability at glutamatergic synapses onto enkephalin-positive (putative D_2_-containing) MSNs. Rats were exposed to the same pretreatment regimen of EtOH, nicotine, or vehicle that resulted in tolerance to EtOH-induced motor impairment and increased EtOH self-administration. The day after the last drug administration, coronal slices were taken for electrophysiological recordings (Fig. 4*A*). Recordings were restricted to the DLS, and stimulating electrodes were placed between the recording electrode and the corpus collosum (Fig. 4*B*) in order to preferentially stimulate cortical inputs. Recording pipettes were also filled with biocytin, so that we could later visualize the MSNs from which we made recordings. Immunohistochemistry for enkephalin was performed on these neurons, and all neurons included in our analyses were enkephalin-positive MSNs (Fig. 4*C*). Both EtOH and nicotine pretreatment resulted in increased paired-pulse ratios at these DLS synapses compared to vehicle pretreatment (Fig. 4*D*,*E*; one-way ANOVA, *p* < 0.0001; Holm–Sidak test for multiple comparisons: *p* < 0.0001, EtOH vs vehicle; *p* = 0.0454, nicotine vs vehicle). EtOH pretreatment also increased the PPR compared with nicotine pretreatment (*p* = 0.0017). This increase in paired-pulse ratio suggests that EtOH and nicotine pretreatment both decrease the probability of glutamate release at D_2_-containing MSNs in the DLS.

**Fig. 5.**
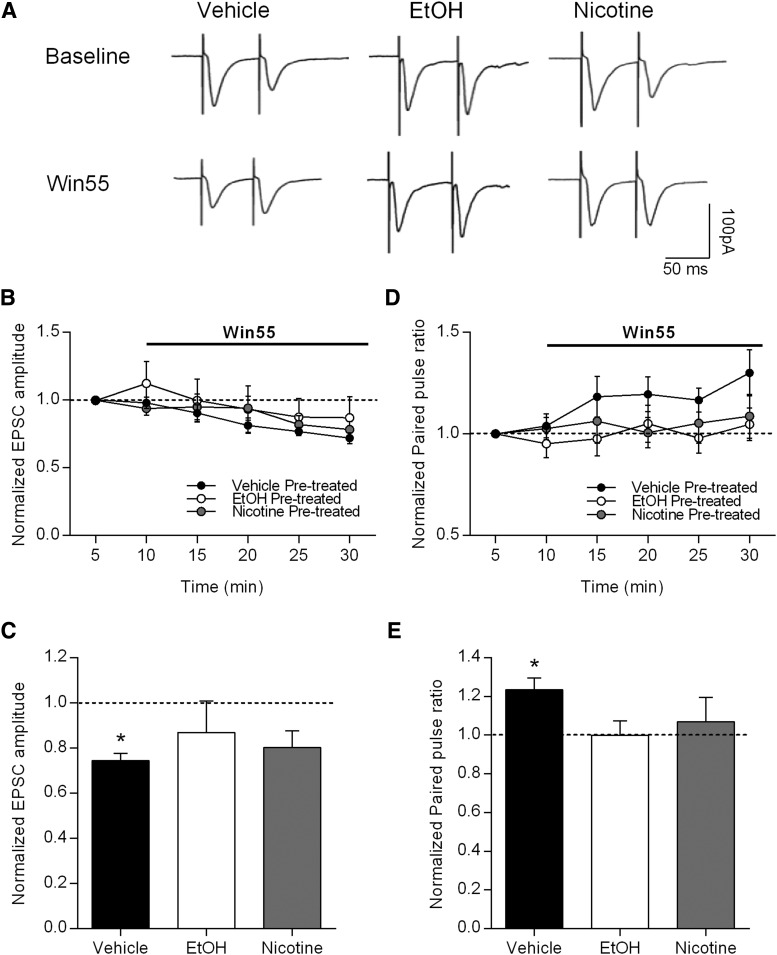
Nicotine or EtOH pretreatment occludes the effects of the CB1 receptor agonist on synaptic transmission in enkephalin-positive MSNs in the DLS. ***A***, Representative traces of evoked EPSCs in putative D_2_-containing MSNs from rats that had been pretreated with vehicle, EtOH, or nicotine during baseline and during application of the CB1 receptor agonist Win 55,2-212 (5 μm). ***B***, Time course for the effects of Win 55, 2-212 on evoked EPSC amplitudes in each of the pretreatment groups. ***C***, Summary data showing that the inhibitory effect of Win 55, 2-212 was only present in vehicle pretreated animals (paired *t* test baseline vs Win 55, 2-212, *p < 0.05; vehicle, *n* = 6; EtOH, *n* = 7; nicotine, *n* = 5). ***D***, Time course for the effects of Win 55, 2-212 on PPR in each of the pretreatment groups. ***E***, Summary data showing that Win 55, 2-212 only increases the PPR in vehicle-pretreated rats (paired *t* test baseline vs Win 55, 2-212, *p < 0.05; vehicle, *n* = 6; EtOH, *n* = 7; nicotine, *n* = 5).

This change in release probability is consistent with a role for endocannabinoid signaling in the DLS. If the effects of EtOH and nicotine pretreatments on PPR were due to changes in endocannabinoid signaling, then the effects of bath application of a CB1 receptor agonist might be occluded in these pretreated rats. To further investigate the potential role of DLS cannabinoid signaling in cross-tolerance between EtOH and nicotine, we again used slice electrophysiology and tested the effects of a bath-applied CB1 receptor agonist (Win 55,2-212, 5 µm). Rats were given the same pretreatments as in all prior experiments of vehicle, EtOH, or nicotine. In vehicle-pretreated rats, bath application of Win 55,2-212 significantly decreased the amplitude of evoked EPSCs, but in EtOH- and nicotine-pretreated rats, Win 55,2-212 application failed to alter EPSC amplitude [Fig. 5*A–C*; paired *t* test comparing 5 min of baseline with the last 10 min of Win 55,2-212 application within each treatment group: vehicle (*n* = 6), *p* < 0.001; EtOH (*n* = 7), *p* = 0.384; nicotine (*n* = 5), *p* = 0.052].

We also examined the effects of Win 55,2-212 application on PPRs in the three treatment groups. While Win 55,2-212 application increased the PPR as expected in vehicle-pretreated rats, the effects of Win 55,2-212 were indeed occluded in both EtOH- and nicotine-pretreated rats [Fig. 5*A*,*D*,*E*; paired *t* test comparing 5 min of baseline with the last 10 min of Win 55,2-212 application within each treatment group: vehicle (*n* = 6), *p* = 0.012; EtOH (*n* = 7), *p* = 0.992; nicotine (*n* = 5), *p* = 0.611]. These data suggest that EtOH and nicotine pretreatment may have enhanced cannabinoid release and thus decreased CB1 receptor expression. These results indicate that pretreatment with either EtOH or nicotine results in similar modulation of endocannabinoid signaling in the DLS, and that this modulation may contribute to cross-tolerance between these two commonly abused drugs.

### Nicotine pretreatment occludes HFS-induced LTD

**Fig. 6.**
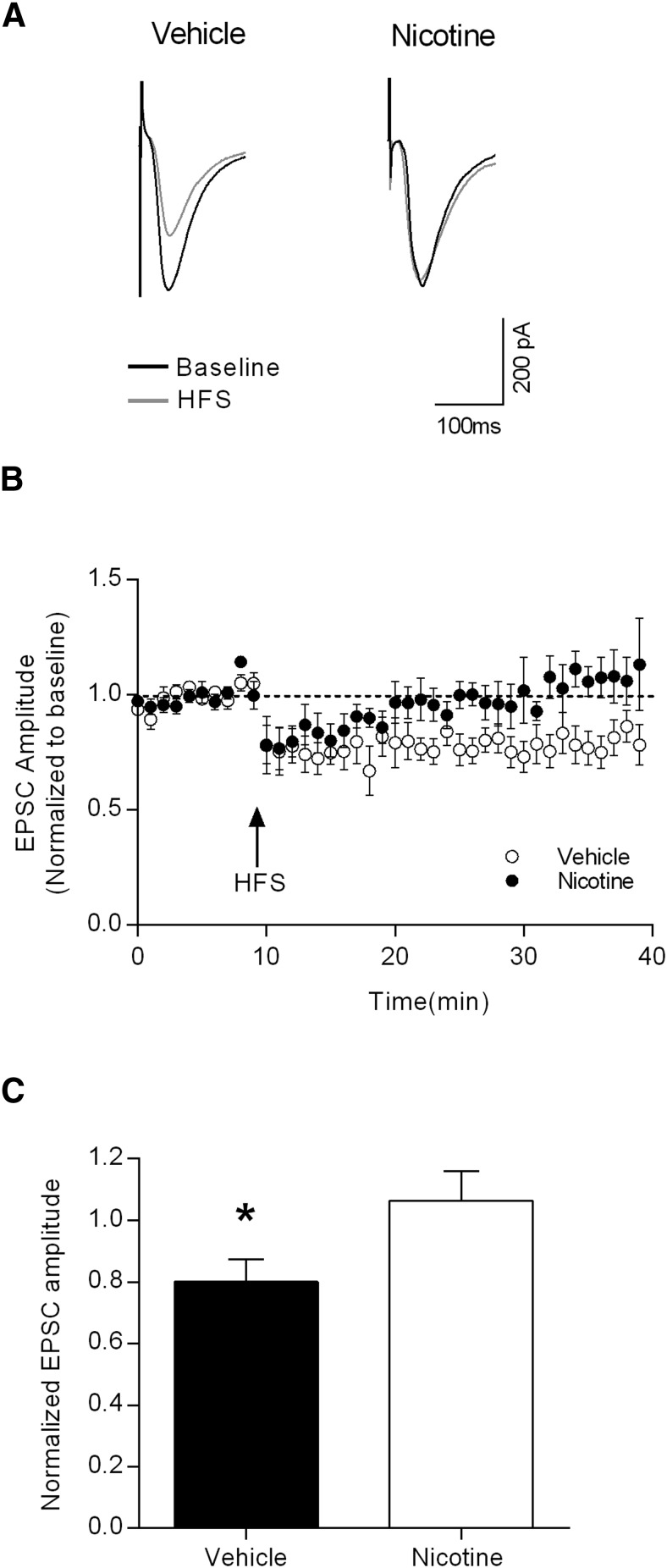
Nicotine pretreatment occludes HFS-induced LTD in enkephalin-positive MSNs in the DLS. ***A***, Representative EPSCs from putative D_2_-containing MSNs from vehicle- and nicotine-pretreated rats during baseline and after HFS. ***B***, Time course of the effects HFS on evoked EPSC amplitudes in vehicle- (open symbols) and nicotine- (filled symbols) pretreated groups. ***C***, Summary data showing that HFS induces LTD of the excitatory inputs to DLS MSNs in vehicle-pretreated rats, but not in nicotine-pretreated rats (unpaired *t* test baseline vs HFS, *p < 0.05; vehicle, *n* = 9; nicotine, *n* = 7).

To further examine the extent to which DLS synaptic plasticity underlies the observed behavioral effects of nicotine pretreatment, we tested LTD induction at corticostriatal synapses. Using the same stimulating electrode placement as that shown in Figure 4*B*, baseline EPSCs were recorded for 10 min, followed by a 1 s HFS train (100 Hz). EPSC amplitudes were then monitored for 30 min. As shown in the raw traces in Figure 6*A* and the time course data in Figure 6*B*, nicotine-pretreated animals showed less LTD following HFS than that observed in vehicle-pretreated rats. Comparing the averaged normalized EPSC amplitudes between the groups revealed significant occlusion of LTD induction in the nicotine-pretreated animals (Fig. 6*C*; *p* < 0.05 with unpaired *t* test).

## Discussion

In this study, we have found that identical pretreatments with either nicotine or EtOH result in both tolerance to the motor-impairing effects of EtOH and increased EtOH self-administration. These pretreatments also result in an increase in the paired-pulse ratio of evoked EPSCs and the occlusion of the effects of a CB1 agonist in putative D_2_-containing MSNs in the DLS. In addition, nicotine pretreatment also occluded HFS-induced LTD at these synapses.

The effects of nicotine on EtOH-related behaviors are rarely examined without the concurrent administration of both drugs. Here we show that nicotine pretreatment alone results in increased EtOH self-administration, despite termination of nicotine administration 24 h prior to EtOH access. We also show that identical nicotine pretreatment results in tolerance to the motor-impairing effects of EtOH, suggesting that increased tolerance to EtOH leads to more EtOH consumption. That tolerance to EtOH and dependence go hand in hand is not a new idea (Pava and Woodward, 2012); however, we used identical pre-exposure paradigms to more conclusively examine how self-administration and tolerance relate to each other.

Nicotine was used to induce tolerance to EtOH, which eliminates a confounding variable that is often built into experiments that attempt to explore this relationship by testing both drugs together. Our finding that nicotine pre-exposure produces tolerance to EtOH and is sufficient to enhance EtOH self-administration suggests that tolerance to EtOH is not simply a byproduct of increased EtOH consumption, but rather that tolerance to EtOH can precede and result in increased EtOH consumption. These findings suggest that a limited history with nicotine can impact EtOH-related behaviors even after nicotine exposure has ended. In human subjects, smoking history correlates with future EtOH dependence (John et al., 2003; Buu et al., 2014). Our findings suggest that prior nicotine exposure in humans may facilitate the development of EtOH tolerance and habitual EtOH consumption.

This idea is supported by past studies in humans, which found correlations between decreased sensitivity to the effects of EtOH and increased risk for alcohol use disorders (Trim et al., 2009; Corbin et al., 2013). Repeated exposures to EtOH may also selectively reduce the experience of the more aversive effects of EtOH (cognitive/motor impairments), while leaving the rewarding effects less affected or even sensitized (Ding et al., 2012; Fritz et al., 2014). Despite the complexities underlying tolerance and sensitization (Blomqvist et al., 1996; Söderpalm et al., 2000; Cohen et al., 2002; López-Moreno et al., 2008), our findings support the hypothesis that cross-tolerance between nicotine and EtOH contributes to the frequent coabuse of these drugs.

In addition to exploring the behavioral outcomes of EtOH and nicotine pretreatments, we also tested neuroplasticity changes in the DLS, as neuroplasticity is known to be important for habit formation, motor performance, and motor learning. Therefore, we tested the impact of EtOH or nicotine pretreatment on plasticity that influence locomotion and drug pursuit. We show that pretreatment with either nicotine or EtOH induces changes in paired-pulse ratio in recordings from putative D_2_-expressing MSNs in the DLS that are consistent with the induction of LTD. This interpretation is supported by the lack of effect of cannabinoid receptor agonist on EPSC amplitude and P2/P1 ratio in striatal brain slices from nicotine- or EtOH-pretreated rats. We further show that high-frequency stimulation of corticostriatal inputs to the DLS, which induced LTD in slices from vehicle-treated animals, did not induce LTD in slices from nicotine pretreated rats. Our working model is that nicotine induces LTD at the excitatory inputs to D_2_-expressing MSN in the DLS and occludes subsequent LTD induction, either by CB1 agonist or HFS. These data suggest that nicotine may induce tolerance to the motor-impairing effects of EtOH and increase EtOH self-administration through changes in corticostriatal synaptic plasticity. These correlative findings support the model that the DLS contributes to drug reward and habit formation (Gerdeman et al., 2003), and future studies will be required to establish causal connections.

Although we placed our stimulating electrode close to the border between cortex and the DLS to preferentially stimulate corticostriatal inputs, it is possible that thalamostriatal projections are also active under these conditions. As reported by Wu et al. (2015), frequency-dependent synaptic plasticity and endocannabinoid expression are stronger and more prevalent in corticostriatal relative to thalamostriatal inputs to the DLS. Corticostriatal synaptic plasticity is thought to underlie motor learning and behavioral plasticity, while thalamostriatal inputs carry sensory information to encode salience and facilitate attentional focus. While our stimulation paradigm does not rule out the sampling of thalamic inputs, both the paired-pulse ratio and HFS-induced LTD measurements preferentially assess the effects of nicotine and EtOH on corticostriatal inputs, and we contend that these modifications are relevant to the behavioral end points examined here.

Previous work has implicated endocannabinoid signaling in tolerance to both EtOH and nicotine and in the rewarding effects of both drugs (Basavarajappa, 2007; Hungund and Yaragudri, 2009; Gamaleddin, 2015). EtOH and nicotine both cause hyperactive endocannabinoid signaling, which results in a downregulation of CB1 receptors in the striatum (Basavarajappa et al., 1998; Cohen et al., 2005). These changes in endocannabinoid signaling have also been linked to LTD at corticostriatal synapses (Gerdeman et al., 2002; DePoy et al., 2013, 2015), which is consistent with our findings. CB1 antagonism or genetic deletion reduces or eliminates the effects EtOH or nicotine pretreatment on EtOH self-administration (Poncelet et al 2003; Thanos et al 2005). Together, these findings support the idea that the CB1 receptor system may provide target for interfering with high EtOH consumption and its impact on the progression to addiction.

Many drug exposure paradigms used in those related studies were of longer duration than the pretreatment used here; therefore, our findings highlight the limited number of drug exposures required to cause dysregulation of endocannabinoid signaling in the DLS and profound behavioral changes. Additionally, the effects of nicotine exposure on endocannabinoid signaling have never been directly compared with those of EtOH. That the same pretreatment with either drug results in similar behavioral outcomes and similar neuroplasticity changes suggests that endocannabinoid signaling in the DLS is critical in mediating cross-tolerance between and frequent coabuse of EtOH and nicotine.

## Synthesis

The decision was a result of the Reviewing Editor Christian Lüscher and the peer reviewers coming together and discussing their recommendations until a consensus was reached. A fact-based synthesis statement explaining their decision and outlining what is needed to prepare a revision is listed below. The following reviewers agreed to reveal their identity: Selena Bartlett, Manuel Mameli

I can commend the authors on a nice revision and it is now suitable for publication.
